# Climate variability impacts on sanitation services in an informal urban settlement of Tandale Ward in Dar es Salaam Tanzania

**DOI:** 10.1016/j.heliyon.2025.e42686

**Published:** 2025-02-14

**Authors:** Anna Mremi, Richard Kimwaga, Deogratias M.M. Mulungu, Fides J. Izdori

**Affiliations:** a Department of Water Resources Engineering, College of Engineering and Technology, University of Dar es Salaam, Tanzania; b Department of Water Supply and Sanitation Engineering, Water Institute, Dar es Salaam, Tanzania

**Keywords:** Climate variability index, Drought–flood, Lowland areas, Faecal sludge management services, Onsite sanitation systems, Unplanned settlement

## Abstract

Global efforts to expand access to sanitation are challenged by climate variability, which disrupts Faecal Sludge Management Services (FSMs) including pit/septic tank emptying operations, transportation, treatment processes at drying beds and treatment plants, routine operation and maintenance of facilities, and safe disposal from onsite sanitation systems. This study conducted in Tandale Ward, a low-lying area with informal settlements explores the impact of rainfall variability on faecal sludge management. Analysis of rainfall data revealed significant variability, with an annual CV of 28.08 %, 31.8 % in long rains, 47.0 % in the dry season, and 51.4 % in short rains. The short rain season showed irregular rainfall distribution, with PCI values exceeding 20 % in 1991, 2002, and 2008. SAI analysis highlighted anomalies, particularly a strong positive SAI in 2017 (2.9), an exceptionally wet year. The Mann-Kendall test revealed a moderately increasing trend in annual and seasonal rainfall, with a significant rise during the dry season. High PCI values are associated with flooding, potentially leading to overloaded containment systems, frequent latrine overflows, and contamination of water sources. Conversely, low SAI values are related to prolonged droughts that reduce water availability, disrupting faecal sludge emptying, transportation, and treatment operations. These findings underscore the importance of integrating climate data into sanitation planning to enhance the sustainability and resilience of FSM systems.

## 1 Introduction

Climate change (CC) and variability are some of the biggest challenges that the world is currently facing in the 21st century [1,2]. By the end of the 21st century, 10 million people will be affected by coastal flooding each year due to expected temperature changes between 0.3 and 5 °C [3,4]. Globally, floods, storms, and drought have affected 4.2 billion people (i.e., 95 percent of all people affected by disasters) and caused an equivalent of US$1.3 trillion of damage (i.e., 63 percent of all damage) [3]. By 2050, over half of the world's population will live in water-stressed regions, with 1.6 billion people at risk of floods, requiring an investment of $6.7 trillion by 2030 and $22.6 trillion by 2050 to address the resulting water pollution and related health issues [5,6]. Flooding is becoming a common phenomenon, brought on by prolonged and severe rains as well as several non-climatic factors such as poor urban planning and inadequate or poorly maintained drainage systems [4,5]. Climate variability impacts are already occurring even more quickly than predicted whereby Tanzania is predicted to become warmer by 2.5°–4.5 °C during the year 2080, and experience variability in all rainfall seasons, and coastal erosion [6,7]. The future projection of climate variability puts more stress on planners, policymakers, and the community on how to prepare, respond, and recover from climate variability impacts [8,9]. Thus, SDG 13 target 13.1 insists on strengthening resilience and adaptive capacity to climate-related hazards and natural disasters in all countries. By 2030, the estimated net economic costs of mitigating the effects of climate change in Tanzania are projected to reach between 1 and 2 percent of the country's GDP annually [10].

**Fig. 1:**
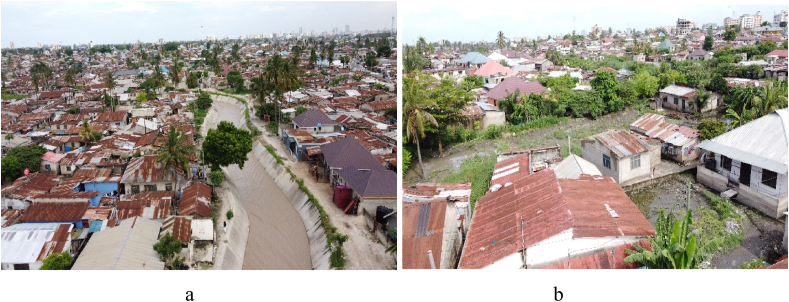
Proximity of settlement from Sinza River (a)and Mzinga River (b) (field data 2023).

It is reported that most of the areas in Dar es Salaam city are unplanned and inhabited by more than 70 % of all people in the city with the challenges of poor faecal sludge management services(FSM) [11]. The provision of new or even maintaining current infrastructure and services is extremely difficult for city and municipal authorities due to population influx and climate-related hazards. About 70–80 % of people in Dar es Salaam City are living in informal areas with inadequate stormwater drainage systems Fig. 5, Fig. 11 and sanitation systems. However, climate variability posed negative effects on sanitation service and infrastructure hindering proper collection, transportation, and disposal of faecal sludge(FS) from onsite sanitation [12,11]. Discharges and polluted overflow from uncontrolled FS and wastewater represent a major source of biological pollution of ecosystems, water bodies, and soil [13]. Effective sanitation systems are essential, especially in densely populated urban areas where the volumes of excreta and the likelihood of exposure increase the hazards associated with improper disposal [14,15]. As shown in Fig. 1, the proximity of people's residences to rivers increases flood risk, especially during the rainy seasons. Therefore, the research aimed to examine climate variability and its implication on faecal sludge management services in the Tandale ward.

## 2 Methodology

### 2.1 Description of study area

**Fig. 2:**
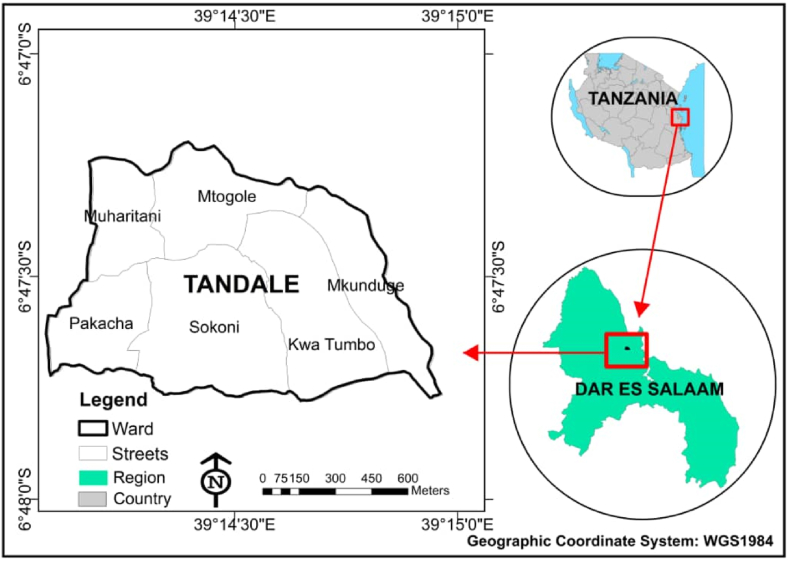
The location of Tandale ward in Kinondoni district (ArcGIS 10.4).

Tandale ward in Fig. 2, is a low-lying area with a Coverage area of 1.159 km^2^ and a population density of 37,464 people/km^2^. This density significantly exceeds the UN-Habitat's sustainable urban density recommendations, which suggest a range that enables efficient public transport and service delivery while maintaining quality of life [16]. The ward is considered overpopulated since its population density is high, with low living standards, and inadequate resources to sustain its population [17]. Tandale's vulnerability is intensified by a high drainage density, causing an increase in surface runoff from numerous parts of the city as people’s settlements are constructed very close to the river as shown in Fig. 1, and flood plains [18]. Stormwater from the upstream areas of Mbezi and Kimara increases runoff and flooding in this ward, which is brought by seasonal rivers called Sinza River and its main tributaries, Mzinga River (Mtunduge) and Kiboko River. Like many other catchments, Sinza River catchment is experiencing significant urbanization upstream which reduces the number of permeable surfaces hence increases runoff [19]. Climatically, the ward has a bi-modal rainfall distribution, with two main rainy seasons long rains and short rains. The long rains season typically occurs from mid-March to the end of May with peak rainfall in April, while the short rains occur from mid-October to late December. The dry season occurred from June-to September. This area is marked by tropical weather conditions, maintaining high temperatures and humidity levels year-round. Daily average temperatures range from 26 °C during the cooler months (June–September) to 35 °C in the hotter season [20].

### 2.2 Material and method

#### 2.2.1 Research design

**Fig. 3:**
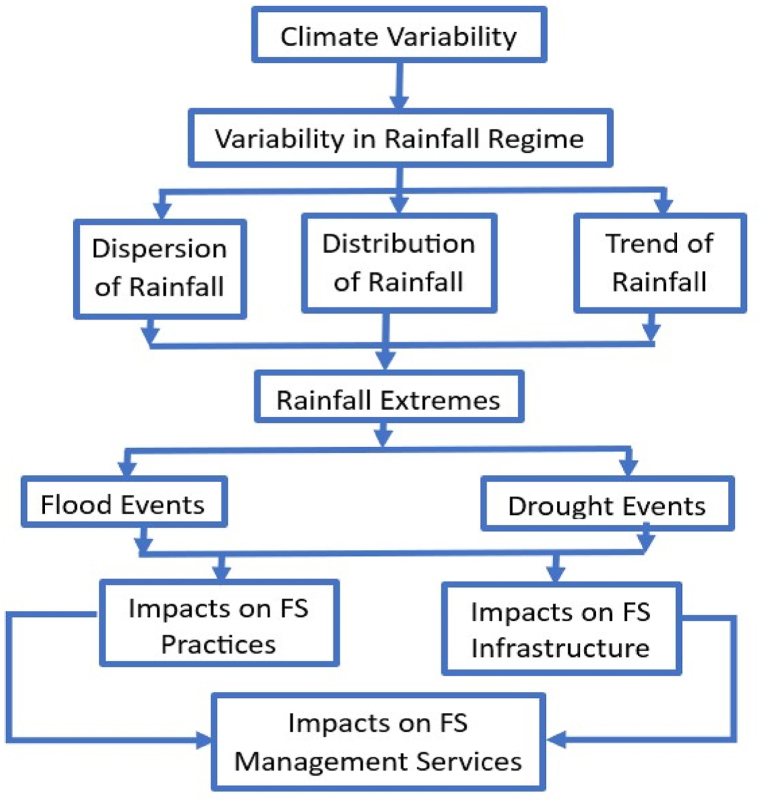
Conceptual diagram of impacts of climate variability on Faecal Sludge (FS) Management.

The study's methodological framework, shown in Fig. 3, indicates detailed steps for data acquisition and analysis for climate variability using rainfall data. The study likewise examines Tandale's faecal sludge management services, focusing on the practices and infrastructures associated with collecting, emptying, transporting, and disposing of faecal sludge. These include septic tanks, roads, pit latrines, and emptying.

#### 2.2.2 Methods for data acquisition

In evaluating climate variability impacts on faecal sludge management services such as the construction of pits and septic tanks, Site and material selection, emptying methods and schedule, accessibility and transportation, and disposal both onsite and onsite. Historical precipitation data for Tandale were required but unfortunately, there are no nearby meteorological weather stations with complete and reliable observed precipitation data. The records at some nearby weather stations, like Ubungo Maji, University College of Dar es Salaam, and Tanganyika Packers, are unreliable, insufficient, and contain discrepancies [21]. Therefore, this study employed observed rainfall data from 1991 to 2017 from the Dar es Salaam International Airport (DIA) weather station, located 15.3 km from Tandale. According to precipitation data from the Tanzania Meteorological Agency (TMA) for Dar es Salaam weather stations, only Port Station and DIA had complete rainfall records. The ground-based observed rainfall data sets are becoming scarcer, mostly in developing countries due to a lack of resources [22]. The World Metrological Organization (WMO) recommends that the standard data needed to undertake climatological investigations be at least 30 years old [23]. Additionally, the analysis utilized CHIRPS satellite daily rainfall data for DIA, which were obtained at a spatial resolution of 4.8 km from climateengine.org. The CHIRPS data were obtained since the rainfall distribution in Dar es Salaam varies [24] and there was no nearby station with complete observed data for Tandale. Pearson correlation is a statistical method employed to quantify the strength and direction of the linear relationship between the variables as it is widely acknowledged as a reliable technique [18,19]. Subsequently, annual and seasonal relationships between CHIRPS and observed data were analyzed and presented in Table 4 and Fig. 5.

Satellite-derived or climate reanalysis products can be utilized as alternatives in places where rainfall data are scarce or absent, even though the rainfall data may have biases and inaccuracies related to sampling, estimating techniques, and observations [18,20]. A growing number of researchers recommend the utilization of Climate Hazards Group InfraRed Precipitation with Station (CHIRPS) data due to its (i) extensive time series, (ii) relatively high spatial resolution, and (iii) free accessibility [22]. Moreover, the CHIRPS data product has undergone a process to address bias and inhomogeneity [22,23]. Over time, CHIRPS has been considered reasonably consistent since it utilizes successful thermal infrared (TIR) from precipitation products like the National Oceanic and Atmospheric Administration’s (NOAA’s) Rainfall Estimate (RFE2) and African Rainfall Climatology or the University of Reading’s TAMSAT African Rainfall Climatology and Time series (TARCAT) [25]. It also estimates and incorporates the mean bias removal process using a satellite-enhanced station-based climatology (CHPclim). This consistency is crucial for long-term climate studies, as it suggests that the data is reliable and has been processed to reduce temporal biases. The CHPclim approach involves blending satellite-derived data with station-based climatological information [25]. It indicates that efforts have been made to correct biases using a combination of both satellite and ground-based observations, enhancing the reliability of the data. Moreover, CHIRPS products have taken steps to mitigate inhomogeneity that may be present in parts of the world where station data availability is inconsistent over time [25]. The blending of station data with the CHIRPS background helps to address inhomogeneities, contributing to a more homogeneous dataset [22].

### 2.3 Acquisition of FSM data

A household survey, literature review, and interview were employed in collecting data to analyze climate change and variability impact on faecal sludge management. The household survey was conducted for 308 households in five sub wards Mtogole, Kwatumbo, Pakacha, Mhalitan, Sokoni, and Mkunduge.

### 2.4 Method for data analysis

The statistical data collected from a household survey conducted in Tandale were analyzed using IBM SPSS statistics 20 and Excel while other qualitative data were presented using photos. The Coefficient of Variation (CV) evaluates seasonal and annual variability the Standard Anomaly Index (SAI) provides light on short-term deviations from the mean, and the Precipitation Concentration Index (PCI) measures the annual distribution of rainfall and highlights the risk of floods or droughts. The Mann-Kendall test looks for trends in rainfall over time, while the Standard Precipitation Indices (SPI) characterize the severity of a meteorological drought [1,23]. Once combined, these methods offer a broad evaluation of rainfall variability that captures dynamics associated with time and trends, strengthening the analysis's reliability and dependability.

#### 2.4.1 Rainfall fluctuations

Standard Anomaly Index (SAI) has been used to calculate the negative and positive anomalies using equation (1). By identifying dry and wet years and their severity levels with the established limits, SAI analysis gives light on the variability of rainfall anomalies during the analyzed period.

(1) $$Z=\frac{Xi-\mu }{S}$$

Where Z is the standardized rainfall anomaly; x_i_ is the annual rainfall for the year I; μ is the mean of annual rainfall and S is the standard deviation of the annual rainfall for the historical observation of the time series while Table 1 gives the classification of SAI according to Ref. [26] of droughts are in three categories: severe, extreme, and moderate.

**Table 1:** The classification of Standard Anomaly Index (SAI) values.

SAI Value	Category
>2	Extremely wet
1.5 to 1.99	Severely wet
1.0 to 1.49	Moderately wet
−0.99 to 0.99	Nearly normal
−1.0 to −1.4	Moderately dry
−1.5 to −1.99	Severely dry
< −2	Extremely dry

#### 2.4.2 Seasonal and annual rainfall variability

Rainfall data was analyzed seasonally and annually over the observed time, and results were divided into high, intermediate, and low categories. A higher CV indicates greater rainfall variability within the study area, whereas a lower CV shows less variability as indicated in Table 2. The coefficient of variation (CV) Equation (2) [1,24] was used to determine the seasonal and annual rainfall variability.

(2) $$\text{CV}=\frac{\sigma }{\mu }*100$$

Where CV is the coefficient of variation, σ is the monthly/annual standard deviation and μ is the mean precipitation of the recording month/year. Greater variability is indicated by higher CV values as indicated in Table 2.

**Table 2:** Rainfall variability index [1].

High	Intermediate	Low
CV > 30	20 < CV > 30	CV < 20

#### 2.4.3 Rainfall distribution

Seasonal and annual distributions of rainfall were considered in the analysis from the time series whereby threshold limits in Table 3 were used. This was useful for identifying the hydrological risks of floods and drought events in the study area [26]. The precipitations concentration index (PCI) Equation (3) [27] was used to calculate rainfall distribution.

(3) $${PCI}_{12}=\frac{\sum_{i=1}^{12}{Pi}^{2}}{{\left(\sum_{i=1}^{12}Pi\right)}^{2}}*100$$

(4) $${PCI}_{3}=\frac{\sum_{i=1}^{12}{Pi}^{2}}{{\left(\sum_{i=1}^{12}Pi\right)}^{2}}*25$$

Where, Pi is the monthly precipitation of the year, the numerical value 100 in equation (3) is to account for the 12 months (100 %) in the annum. Similarly, 25 values in equation (4) are for 3 months in the Long rain, Short rain*,* and dry seasons.

**Table 3:** Categories for Precipitation Concentration Index (PCI).

Precipitation	Uniform distribution	Irregular distribution	Moderate concentration	Strong irregular distribution
PCI	<10	16–20	11–15	>20

**Table 4:** The Pearson correlation of re-analysis data for CHIRPS at DIA.

Pearson Correlation coefficient(r)			
Variables	Annually	Monthly	Seasonally
Uncorrected CHIRPS & Observed	0.81	0.86	0.99
Corrected CHIRPS & Observed	0.83	0.86	0.99

#### 2.4.4 Characterization of extreme events

The characteristics of the meteorological drought basically can be calculated in 1, 2, 3, 6, 18, or 48 months but for this study, it was calculated in 12 months or annual periods. The standard precipitation indices (SPI) equation (5) below was used in this calculation [1,28]

(5) $$\text{SPIij}=\frac{\text{Xij}-\mu \text{ij}}{\alpha \text{ij}}$$

Where the SPI_ij_ represents an ith month at the jth period, Xij is the observed rainfall total value for the ith month at the jth period, μij and, αij represent the long-term mean and standard deviation of the ith month, and jth timescale of the selected period respectively. According to Ref. [29], the categories Table 3 have been used for this analysis.

#### 2.4.5 The Mann- Kendall (MK) trend test and Sen's estimator

Mann-Kendall (Z) test was employed in trend analysis for rainfall data to evaluate the existence and importance of trends from 1991 to 2017. The Sen's slope (Q) estimator is a nonparametric method used to determine the true slope of an existing trend, typically observed as a yearly change [23,25]. It is applicable when a linear trend can be assumed. The MK method was selected over other methods like Spearman’s Rho because it can be used to determine a monotonic trend in a time series with no seasonal or other cycles [16,23,26]. The missing values are acceptable, and there is no need for the data to have a specific distribution. Also, outliers or single data errors have minimal impact on Sen's method. According to Refs. [1,27], the Mann-Kendall value shows the strength and direction of the trend whereby the positive values indicate an increase for that month or season, and negative values indicate a decrease in average measurements. The test involves two hypotheses null hypothesis of no trend Ho, against the alternative hypothesis Hi (increasing or decreasing monotonic trend).

## 3 Results

### 3.1 Result for climate variability

**Fig. 4:**
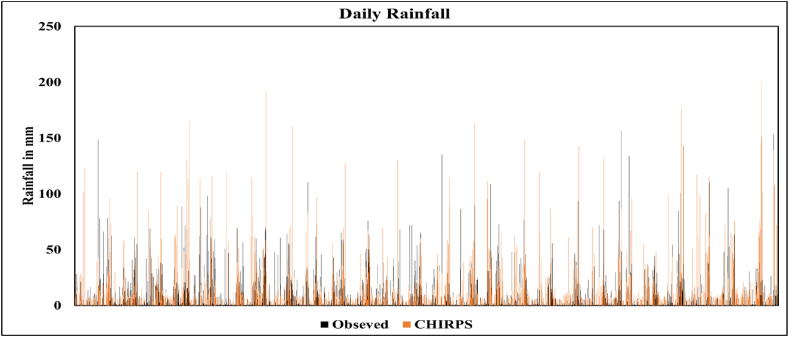
Daily rainfall data for DIA from 1991 to 2017.

To assess the relationship between these two datasets, the daily rainfall data for the CHIRPS and the observed data for the Dar es Salaam International Airport (DIA) station were used, as shown in Fig. 4 daily rainfall data for CHIRPS data and Observed data, demonstrating strong temporal coherence between the two datasets. Both datasets show similar trends; rising and falling together throughout the period. When CHIRPS data showed an increase in rainfall intensity, a corresponding increase was observed in the DIA station data, confirming the reliability of CHIRPS data for rainfall analysis in this region. This correlation validates the use of CHIRPS data for areas where ground-based observations might be limited or unavailable in the study area.

### 3.2 Annual and seasonal correlation between observed and reanalysis data

Daily rainfall data from 1991 to 2017 from both datasets, as shown in Fig. 4 were analyzed to generate monthly, seasonal, and annual data. These data were then used to perform the correlation analysis, as the result is presented in Table 1. A Pearson correlation analysis was performed using Microsoft Excel whereby the strength and direction of the relationship between the two datasets were analyzed and presented in Table 4. According to Ref. [28], a correlation coefficient close to 1 indicates a strong relationship between two datasets, while a coefficient that deviates from 1 indicates a weak relationship.

**Fig. 5:**
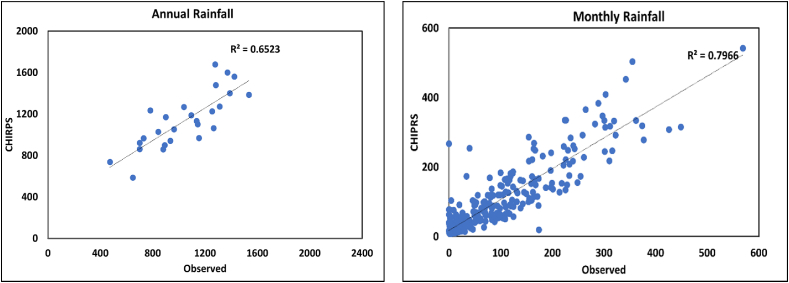
Liner relationship between Observed and Reanalyzed Rainfall data for the years 1991–2017.

The dataset shows a strong positive relationship between the rainfall recorded by the Tanzania Meteorological Agency and the reanalyzed CHIRPS data for DIA. The result shows a strong correlation for monthly, seasonal, and annual data. Despite the similarities in the increasing and decreasing trends of observed and reanalyzed data, the magnitude of the difference is notable, as the linear regression (r^2^) score is lower for annual precipitation data compared to monthly and seasonal data, as presented in Fig. 5. The positive correlation suggests that the two datasets are mutually supportive of one another, improving the overall accuracy and reliability of analyses regarding the fluctuation of monthly rainfall from 1991 to 2017. as indicated in Fig. 5.

**Fig. 6:**
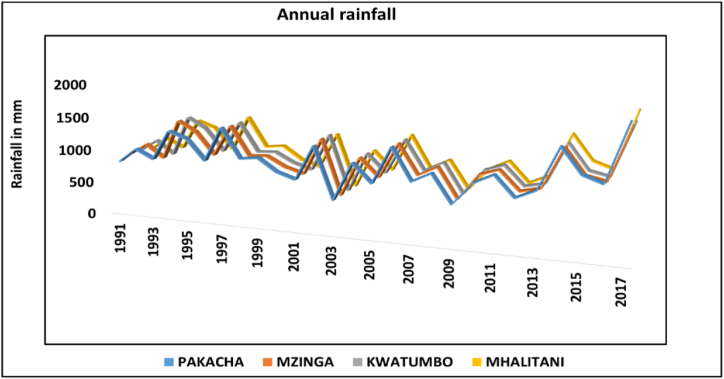
The CHRIPS Rainfall data for four stations generated in Tandale from 1991 to 2021.

The study decided to use CHIRPS data for Tandale since the CHIRPS data exhibit a strong relationship with the DIA's observed data as observed in Fig. 5. To figure out whether there were variances in the amount of CHIRPS rainfall data recorded at various locations in Tandale, this study established four stations in different areas, naming them Pakacha, Kwatumbo, Mzinga, and Mhalitani. Due to the high degree of similarity between the four stations that were created, as shown in Fig. 6, the study opted to use Pakacha's data for the statistical analysis of climate variability.

### 3.3 Descriptive statistics and rainfall distribution (CV)

According to the descriptive statistics of CHIRPS rainfall data recorded from 1991 to 2017 Table 5 shows the average annual rainfall was 1046 mm, the maximum annual rainfall was 1883 mm in 2017, and the minimum annual rainfall was 517 mm, which indicated that 2003 was the driest year during the period. The annual standard deviation was 294 mm, indicating a moderate variability from the mean in rainfall variability. The coefficient of variation (CV) was analyzed in the study giving the CV annual value of 28.08 %, which indicates intermediate annual rainfall variability Likewise, long rain represented a CV of 31.8 %, dry season 47.0 % and Short rain 51.4 % which indicates that rainfall during the short rain season is highly variable and unpredictable, which directly impacts pit latrine emptying operations, increases risks of pit latrine overflow during heavy rains, and affects the soil stability for existing pit latrines. Short rains are particularly unreliable, with rainfall amounts varying greatly from year to year [30] making it challenging for residents to plan and maintain their onsite sanitation facilities. Although a long rain season typically records higher total rainfall, the inconsistent nature and intensity of a short rain season can lead to localized flooding, particularly in poorly drained areas like Tandale Ward.

**Table 5:** Descriptive statistics for annual and seasonal rainfall from 1991 to 2017 For Pakacha.

Time	Mean	Max	Min	SD	CV %	% Annual Contributions
Annual	1046.16	1893.36	517.33	293.74	28 %	100
Long rain	561.05	274.67	265.29	274.67	31.8	54
Dry season	54.55	136.65	25.10	25.66	47.0	5
Short rain	279.46	655.16	15.36	143.59	51.4	27

Where Max represents Maximum, Min represents minimum, SD represents standard deviation, and Cv represents coefficient of variation.

**Fig. 7:**
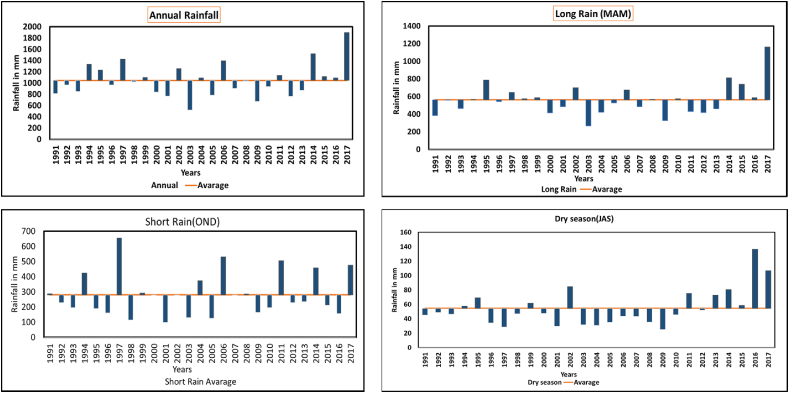
Distribution of Annual and seasonal rainfall from 1991 to 2017 for Pakacha.

The long rainfall season in Fig. 7, indicates the highest rainfall occurs in March, April, and May whereby the highest amount of rain recorded at 1162 mm and the lowest at 265 mm. The data indicates a notable variation in rainfall amounts whereby the year 2003 recorded the lowest rainfall. Depending on the year and particular month with values as high as 1162 mm(2017), the data point to the possibility of major flooding events, especially if the rainfall happens in short, intense periods [31]. Short rainfall season was found to have a wide variation since the average seasonal rainfall recorded was 280 the maximum rainfall was 655 mm recorded in the year 1997 and the minimum rainfall was 15 mm. This time frame is essential for ensuring the restocking of water supplies following the dry season [32]. The uneven distribution of rainfall indicates that water management strategies should be carefully planned because this season might bring both excessive rainfall and dry spells [33]. The dry season has the lowest rainfall which indicates a time when the risk of drought is greater and water availability for flashing toilets and cleanness is of great concern. With minimums as low as 51.25 mm, the data emphasizes the necessity of water conservation techniques and drought management, such as systems to collect and store rainwater in the rainy months for use in the dry season to improve faecal sludge management services, especially in water-dependent systems like poor flash latrines which cannot operate without water.

### 3.4 Annual and seasonal rainfall fluctuations

Results indicate that of the 27 years, 20 years (74 %) had near-normal rainfall and 2 years (7 %) were moderately wet, with the remaining years falling into numerous SAI categories. The most significant rainfall anomaly recorded during the study period occurred in 2017, with an annual anomaly of 2.9 and a long rain anomaly of 3.4, indicating an extremely wet year as shown in Fig. 7. This anomaly caused severe flooding across Dar es Salaam [18], documenting extensive impacts throughout the lower Msimbazi flood plain, including damage to infrastructure and settlements. This anomaly caused flooding, which impacted FSM in Tandale Ward by overwhelming pit latrines and septic tanks, leading to their overflow. The excessive water have also weakened latrine structures, increased contamination risks, and disrupted FSM services due to access challenges and heightened public health hazards [34]. These effects highlight the vulnerability of sanitation infrastructure to extreme rainfall events [35]. 2003 was observed with the lowest amount of rainfall, marked by (SAI value of −1.69) indicating a severe drought. This aligns with previous studies that indicated a widespread drought across several regions of Tanzanian in 2003 [32].

### 3.5 Annual and seasonal rainfall distribution

**Fig. 8:**
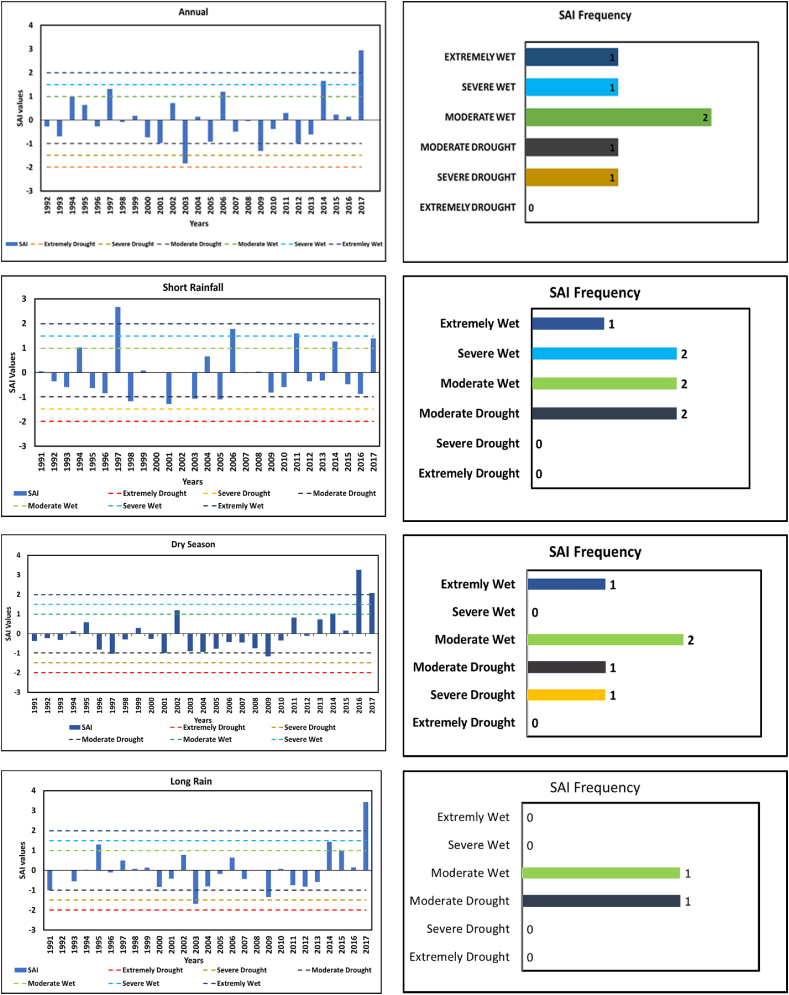
Standard anomaly index showing Rainfall fluctuation.

A higher PCI score indicates a high concentration of rainfall either seasonally or annually. Fig. 8 shows PCI results on an annual and seasonal basis from 1991 to 2027. The long rain season typically shows uniform precipitation in 21 years (77.7 %) with a PCI <10 and 8 years (23.3 %) showing moderate precipitation as per the PCI limit. In 2002 and 2006, PCI values were notably high for long rain this suggests that these years are likely to have significantly high rainfall concentration, which could have led to increased risks of flooding and storm-related impacts as Tandale is a low-lying area. The dry season season demonstrates uniform precipitation, with 23 years (85 %) having a PCI <10. The short rain season is categorized with a strongly irregular rainfall distribution as shown in Fig. 8 below, with high concentrations that occurred over short periods. As documented by this pattern may be linked to floods and erosion. Similar high concentrations of rainfall were observed during the long rain season in 1991, 1994, and 1997, and the dry season in 2010. Notably, short rain was presented with higher PCI >20, indicating a strongly irregular rainfall distribution with higher rainfall concertation in the time series which may lead to floods or drought. This can be considered as a potential to support sustainable planning for water and FSM services include design and construction of water management systems for sanitation.

### 3.6 Rainfall characteristics for extreme events (flood & drought)

**Fig. 9:**
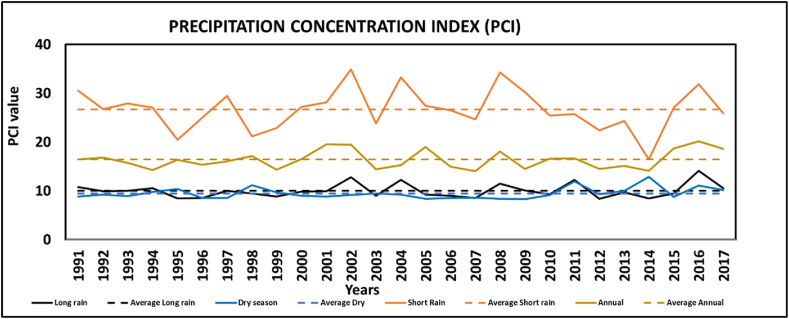
Result for seasonal and annual PCI from 1991 to 2017.

The analysis used annual rainfall data from October 1991 to September 2017 whereby Fig. 9 illustrates the rainfall variation, ranging from wet to extremely dry years, with positive values indicating flooding or wet years and negative values representing drought. Moreover, Table 6 outlines drought events and episodes observed over the 27-years. Two notable drought episodes were recorded the first drought period lasted for 20 months from April 2003 to December 2004 indicating extreme drought with an SPI value of −2.47. The second drought period had a peak SPI value of −2.01 and lasted 25 months from November 2009 to December 2011. The identified extreme drought episodes for such a long time (20 and 25 months) have significant implications for water provision highlighting the need for robust water management strategies and resilience-building measures to address water scarcity during these periods, for example, rainwater harvest as recommended by Ref. [36]. During these extreme events, the implications on FSM are significant, including frequent filling [37] and emptying of containment systems due to higher demands, increased blockages caused by roots seeking water in dry soils [38], and challenges in sludge transportation caused by deteriorated road infrastructure exacerbated by rainfall during transitions from drought to wet periods.

**Table 6:** The SPI (Standardized Precipitation Index) drought period_12_m_-2.

Drought	Start date	End date	duration	peak	sum	average	median
1st period	2003-04-01	2004-12-01	20	−2.47	−29.02	−1.45	−1.65
2nd Period	2009-11-01	2011-12-01	25	−2.01	−24.38	−0.98	−0.9

### 3.7 Rainfall trend

**Fig. 10:**
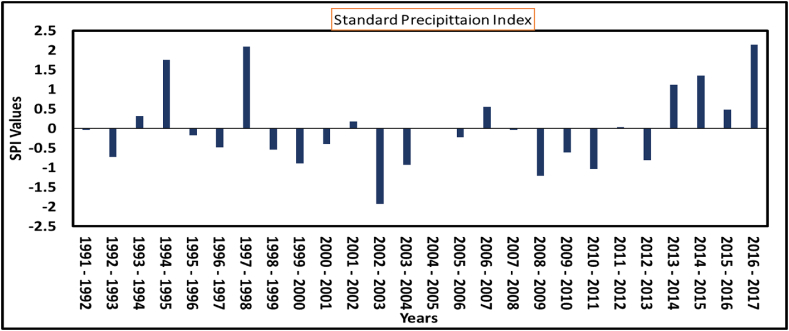
Analysis of extreme weather events using SPI.

**Fig. 11:**
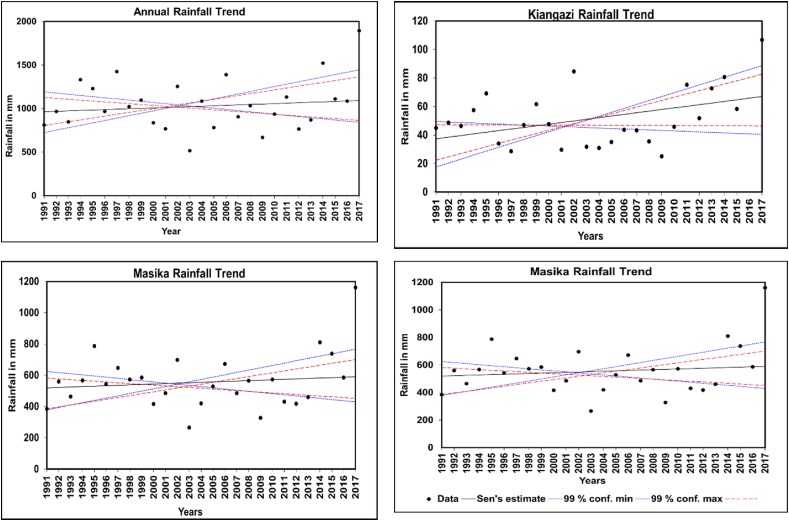
Identified annual and seasonal trends at 99 % confidence level.

For trend analysis positive values indicate an increasing trend, while negative values show a decreasing trend for both Z and Q values. A 99 % confidence level, was used in the trend analysis as presented in Fig. 10. The study in Table 7 demonstrates an increased trend for annual, long rain, and short rain from 1991 to 2017 but is not statistically significant at the 99 % confidence level. The dry season also shows an increasing trend with a trend score of 1.8 and a slope of 1.1, this represents a weak significant increased trend in the 99 % but may be significant in 90 %. However, the trend would be deemed statistically significant at the 99 % confidence level if the Z-score was at least 2.6. Generally, the annual and seasonal rainfall for the data set shows an increasing trend, aligning with studies by Ref. [39]and similar findings by Ref. [40] that reported increasing rainfall trends in Dar es Salaam. These trends have critical implications for FSM in Tandale since the ward is adjacent to rivers such as Sinza, Kiboko, and Mtunduge with elevations ranging from 7 to 44 m above sea level. The increasing rainfall intensifies surface runoff, which is especially problematic for settlements near rivers where pit latrines are vulnerable to flooding, overflow, inaccessibility, and structural damage. This geographical vulnerability, combined with increasing rainfall trends, creates compound risks for FSM infrastructure. Rising groundwater levels can compromise pit stability, while increased runoff can overwhelm and damage existing sanitation facilities. This suggests an urgent need for flood-resistant FSM structures and strategic placement of new facilities away from flood-prone areas (see Fig. 11).

**Table 7:** The Mann-Kendall test and Sen’s slope estimator test.

Time	Mann Kendal test (Z)	Signific.	Sens’ slope (Q)
**Long rain**	1.0		2.7
**Dry season**	1.8	+	1.1
**Short rain**	0.3		0.7
**Annual**	0.6		5.0

+: significantly increasing.

## 4 General discussion of the findings

The analysis reveals a strong positive correlation between observed and reanalyzed rainfall data, aligning with findings from previous research [6,17,19] which highlights the reliability of CHIRPS data. Both annual and seasonal rainfall patterns show substantial variability, consistent with other studies [1,21,23]. This variability, especially during the short rain season, shows irregular rainfall distribution with high concentrations of Precipitation Concentration Index (PCI) values exceeding 20 %. Such patterns may be linked to increased flood risks, as also noted by Refs. [8,41], who observed that regions with high rainfall variability face frequent disruptions in FSM services due to floods and droughts impacting infrastructure and operational capacity. Also [41], reported that rainfall increase in Dar es Salaam has been associated with an intensification in flood incidence, which poses a significant impact on the economy and communities’ livelihoods.

Further, the research indicates that droughts can lead to reduced water availability, negatively affecting sanitation facility operations and concentrating pollutants [30,42] On the other hand, floods, as highlighted by Ref. [43] can cause sanitation systems to overflow, leading to contamination and health risks. Damage to infrastructure is a common consequence, resulting in service disruptions. In the context of Tandale Ward, the study identifies notable variations in annual and seasonal rainfall between 1991 and 2017, with increasing trends where the higher trend value of (1.8 mm) was recorded in the dry season. These fluctuations can saturate the ground, leading to floods in years with higher rainfall such as the year. Such conditions may reduce pit latrine infiltration rates, leading to latrine overflow, damage to stormwater infrastructure, contamination of water sources, and other environmental and public health risks.

Given the global emphasis on expanding access to sanitation, ensuring that FSM services are reliable, accessible, and functional is crucial. The design of FSM infrastructure must integrate local climate variability to guide the formulation of plans, regulations, and guidelines for climate-resilient infrastructure with clear performance goals [44]. With DAWASA's plans to expand sewer and simplified networks in Dar es Salaam, including Tandale, ensuring a reliable water supply for sanitation is essential. Water shortages due to drought may limit the operation of water seal pans, reducing the effectiveness of these sanitation solutions [45]. To address this, FSM systems can be improved by incorporating drought-resilient strategies, such as promoting the use of dry sanitation technologies, enhancing rainwater harvesting systems to ensure water availability during droughts, and establishing decentralized FSM services to reduce reliance on water-dependent systems. Understanding these fluctuations is vital for enhancing sustainability and monitoring sanitation infrastructure, particularly in low-lying communities more susceptible to runoff and floods.

## 5 Conclusion and recommendation

This study evaluated climate variability in Tandale Ward using statistical tools such as CV, PCI, SAI, and the Mann-Kendall test to assess its implications for fecal sludge management (FSM). The findings indicate high rainfall irregularities, particularly during the short rain season, with exceptional PCI values exceeding 20 %, such as in 2002 (34.9 %) and 2008 (34.4 %), which significantly increased flood risks. The SAI highlighted key anomalies, including a strong positive anomaly in 2017 (2.9), marking an exceptionally wet year, and a pronounced negative anomaly in 2003 (−1.8), reflecting drought conditions. Trend analysis using the Mann-Kendall test revealed an upward trend in rainfall, with slopes of (Q = 1.1) for the dry season, (Q = 2.7) for the long rains, and (Q = 5.0) annually, though not always statistically significant.

These findings demonstrate the significant impact of climate variability on FSM services in Tandale Ward. Flooding during high PCI years leads to overloaded containment systems, frequent latrine overflows, and contamination of water sources, while prolonged droughts in low SAI years reduce water availability, hindering faecal sludge emptying, transportation, and treatment operations. To mitigate these challenges, the study underscores the urgency of integrating climate-resilient strategies into FSM infrastructure and operations. The may include; elevated and flood-resistant containment facilities, enhancing road and drainage systems, encouraging water-saving technologies and practices during droughts, and promoting adaptive sanitation planning informed by detailed and continuous climate data analysis.

## CRediT authorship contribution statement

**Anna Mremi:** Writing – review & editing, Writing – original draft, Visualization, Methodology, Formal analysis, Data curation, Conceptualization. **Richard Kimwaga:** Writing – review & editing, Supervision, Conceptualization. **Deogratias M.M. Mulungu:** Writing – review & editing, Supervision, Conceptualization. **Fides J. Izdori:** Writing – review & editing, Supervision, Conceptualization.

## Consent to participate

Authors consent to their participation in the entire review process.

## Availability of data and materials

All relevant data are included in the paper or its Supplementary Information.

## Ethical approval

All work complies with ethical standards.

## Consent for publication

The authors give their permission to publish.

## Declaration of competing interest

The authors declare the following financial interests/personal relationships which may be considered as potential competing interests:

Anna Mremi reports administrative support was provided by University of Dar es Salaam College of Engineering and Technology. If there are other authors, they declare that they have no known competing financial interests or personal relationships that could have appeared to influence the work reported in this paper.
